# Dynamic Warning Method for Structural Health Monitoring Data Based on ARIMA: Case Study of Hong Kong–Zhuhai–Macao Bridge Immersed Tunnel

**DOI:** 10.3390/s22166185

**Published:** 2022-08-18

**Authors:** Jianzhong Chen, Xinghong Jiang, Yu Yan, Qing Lang, Hui Wang, Qing Ai

**Affiliations:** 1 College of Civil Engineering, Chongqing University, Chongqing 400044, China; 2 China Merchants Chongqing Communications Technology Research & Design Institute Co., Ltd., Chongqing 400067, China; 3 State Key Laboratory of Coal Mine Dynamics and Control, Chongqing University, Chongqing 400044, China; 4 Hong Kong-Zhuhai-Macao Bridge Authority, Zhuhai 519060, China; 5 School of Naval Architecture, Ocean and Civil Engineering, Shanghai Jiao Tong University, Shanghai 200240, China

**Keywords:** dynamic warning method, structural health monitoring, ARIMA, Hong Kong–Zhuhai–Macao Bridge, immersed tunnel

## Abstract

Structural health monitoring (SHM) is gradually replacing traditional manual detection and is becoming a focus of the research devoted to the operation and maintenance of tunnel structures. However, in the face of massive SHM data, the autonomous early warning method is still required to further reduce the burden of manual analysis. Thus, this study proposed a dynamic warning method for SHM data based on ARIMA and applied it to the concrete strain data of the Hong Kong–Zhuhai–Macao Bridge (HZMB) immersed tunnel. First, wavelet threshold denoising was applied to filter noise from the SHM data. Then, the feasibility and accuracy of establishing an ARIMA model were verified, and it was adopted to predict future time series of SHM data. After that, an anomaly detection scheme was proposed based on the dynamic model and dynamic threshold value, which set the confidence interval of detected anomalies based on the statistical characteristics of the historical series. Finally, a hierarchical warning system was defined to classify anomalies according to their detection threshold and enable hierarchical treatments. The illustrative example of the HZMB immersed tunnel verified that a three-level (5.5 σ, 6.5 σ, and 7.5 σ) dynamic warning schematic can give good results of anomalies detection and greatly improves the efficiency of SHM data management of the tunnel.

## 1. Introduction

The deterioration of an immersed tunnel is a natural process with the increase in its service time. This may result in extensive deterioration and may affect the tunnel’s serviceability [1,2,3,4,5,6,7,8]. Real-time SHM of the immersed tunnel is an effective way to solve this problem. It can monitor the dynamic changes of a tunnel’s structure by embedding all kinds of monitoring equipment in the tunnel structure during the construction process. Further, the SHM system help to show dynamic responses of infrastructure whenever they are under heavy traffic load together with the obtained parameters depending on the traffic conditions [9]. The collected data from the equipment can then be analyzed and guide refined tunnel operation and management [10,11,12,13,14,15,16].

However, for many years, tunnel operators relied on their judgment or simple fixed alarm threshold to monitor structural data and determine maintenance measures. If this threshold is set too high, it is difficult to carry out an effective warning as the system will not set the alarm bell even if the data behave abnormally. On the other hand, if the threshold is too low, the alarm frequency will be very high, which may lead to resource mismatch. Clearly, the fixed threshold method oversimplifies the issue and results in the low reliability of anomaly detection. Moreover, it does not take into account the aging of the tunnel structure and does not make full use of the massive monitoring data.

As the digital transformation in infrastructure is accelerating, the data-driven anomaly detection method has become a research hotspot. Among them, the regression model based on ARIMA is widely used [17]. The idea is to have the model capture the normal behavior of the time series, whereas a significant deviation from this model is supposed to be an outlier. 

ARIMA is a classic model for time series forecasting in the engineering field. Huang et al. [18] concluded the ARIMA model is capable of predicting the evolution of tunnel deformational performance with low computational cost and satisfying accuracy in the short term. Bian et al. [19] designed a forecasting model on the strain monitoring data of the SHM system of a cable-stayed bridge by combining the empirical mode decomposition method with the ARIMA and showed that the proposed method performs better in all statistical indicators. Liu et al. [20] built an ARIMA model to predict the concrete damage failure of the service tunnels under sulfate erosion. It can be seen that the ARIMA model has been widely applied in predicting the evolution of tunnels and bridges, and it can be combined with other models to achieve a better prediction effect [21,22,23,24,25].

Some researchers in other fields further adapted the ARIMA method to detect anomalies and issue warnings. Li et al. [26] combined the ARIMA model and the transferable belief model to realize the early warning of coal and gas outbursts in coal mining production. Zeng et al. [27] proposed an improved ARIMA model to detect the outliers based on dynamic variance. By selecting an appropriate and adjustable threshold, the algorithm successfully reduced the warning error of optical fiber sensors caused by the normal fluctuations. 

Nowadays, although the government has attached great importance to tunnel maintenance and digital transformation, the research on anomaly detection of the immersed tunnel based on mass monitoring data is still insufficient. This study has aimed to propose a more intelligent and reliable warning method for immersed tunnel structures, which is of great significance to improve the safety of the structure, ensure the safety of the operation, reduce the maintenance cost, forecast disasters, and improve the overall service level. The innovation of this study lies in the design of a dynamic warning method for SHM data based on ARIMA prediction so as to timely and accurately detect anomalies. The model is dynamic, which means it can be automatically updated to capture the sequence trend in real-time. At the same time, the threshold is set to be dynamic so that the local characteristics of the sequence are considered in the process of judging outliers.

The paper is organized as follows: Section 2 will introduce the basic information about the investigated project—the Hong Kong–Zhuhai–Macao Bridge immersed tunnel and its SHM system. After that, the denoising method and the classification of anomalies will be introduced. Section 3 will discuss the methodology, which includes the static ARIMA model, the dynamic ARIMA model, and the anomaly detection mechanism, and apply this method to concrete strain monitoring data to verify the effectiveness of the proposed method. Section 4 gives the concluding remarks.

## 2. Outlines of the Investigated Project

### 2.1. Outlines of the HZMB Immersed Tunnel

**Figure 1:**
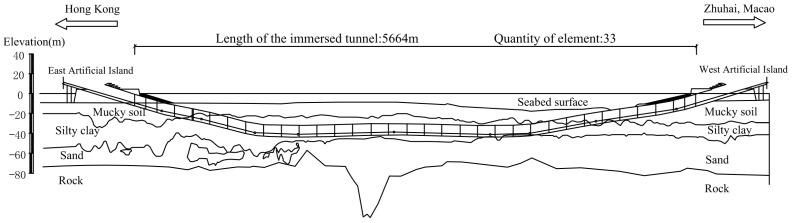
Longitudinal layout of the HZMB immersed tunnel.

The Hong Kong–Zhuhai–Macao Bridge (HZMB), spanning Lingdingyang Bay, is a 55-km-long mega project. It includes three components: the main project of the bridge, island, and undersea tunnel; the ports of Hong Kong, Zhuhai, and Macau; and the connecting line between the three cities. As a part of the main project, the most challenging task is the 6.7-km-long undersea tunnel [28]. The immersed tunnel section has a length of 5.67 km and consists of 33 elements, as shown in Figure 1. Among all the elements, E28–E33 are located on the flat curve with a radius (R) of 5500 m, and the rest are located on the straight section [29]. The closure joint is between E29 and E30, and the water depth at the bottom of the closure joint is 27.9 m.

**Figure 2:**
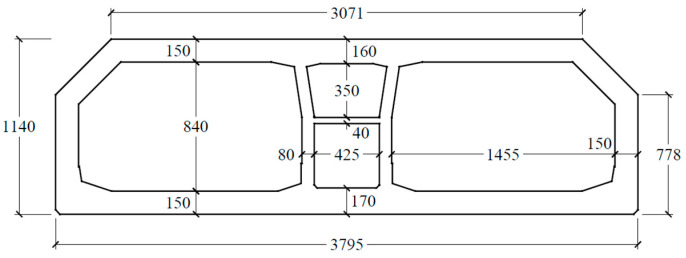
Cross-sectional geometry of the HZMB immersed tunnel.

The standard tunnel element is 180 m long, consisting of eight segments, each of which stretches 22.5 m. The immersed tunnel provides a dual three-lane carriageway with a width of 2 × 14.55 m and a vertical clearance of 8.4 m, as shown in Figure 2.

As a complex sea-crossing project, the HZMB immersed tunnel has a transition section at the head of the artificial islands where different foundation solutions are adopted. Additionally, the thickness of the placed backfill is uneven [30]. Meanwhile, the deposition of sediments should also be taken into consideration during the operation life of the structure, which increases the overlying load on the tunnel structure. Load changes in the tunnel’s longitudinal direction will also result in a larger internal force in the tunnel. All the above situations will make the operational safety of the immersed tunnel’s structure a great challenge.

### 2.2. Overview of Tunnel SHM System

HZMB is a mega infrastructure project with huge investment and significant impact, and a designed life of 120 years. In order to manage the maintenance and ensure the safety of this underwater structure, a SHM system is introduced to the project.

SHM is a comprehensive multidisciplinary system that encompasses techniques in sensing operation, electronic engineering, signal analysis, network communication, computer engineering, pattern recognition, civil engineering, and so on. Its main functions include: (1) Conduct effective monitoring and effective maintenance measures throughout the whole operation process; (2) Develop a system that works coherently in each stage of construction and operation; (3) Provide necessary and practical information for the operation staff on the structural state; (4) Predict the health state of the structure based on the systematic risk analysis and structural response and carry out the corresponding maintenance measures to guarantee structural safety.

According to the above functional requirements, the system includes the following subsystems:

(1) Automatic sensing system, which includes the following three modules. The first is the sensor module. This module serves to control various types of sensing equipment, read structural load data and structural response data, and convert these values into voltage, electric current, or frequency. The second is the data acquisition and transmission module, performing the function of converting the collected electrical signal into a digital signal that can be recognized by the computer and transmitting it to the data processing and control subsystem through the wired and wireless network. The third is the data processing and control module, whose function is to complete data pre-processing, post-processing, archiving, display, and storage.
(2) Inspection and maintenance management system. This subsystem formulates the regulations of structure inspection and maintenance, arranges personnel to carry out periodic, quantitative, standard, and systematic inspections according to the tasks set by the software.
(3) Structural evaluation and early warning system: The main function of this subsystem is to make precise assessments of the structural operation state with the help of high-performance computing equipment, a variety of static and dynamic analysis software, and damage inspection results. Then, provide technical support to the management department by preparing and submitting the monitoring report regularly.
(4) Central database system: This subsystem manages and stores the static information and dynamic monitoring data of the whole monitoring system.
(5) User interface subsystem: Display all kinds of data to users and accept users’ control and input of the system.

**Figure 3:**
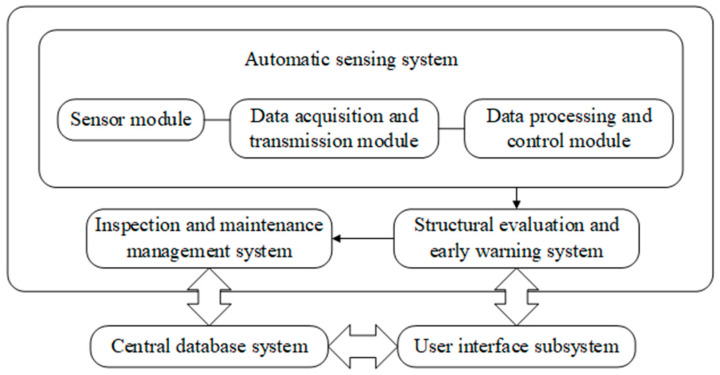
SHM system of the HZMB.

The relationships between subsystems are shown in Figure 3.

Five types of monitoring data, namely ground motion, joint deformation, concrete strain, temperature, and humidity data, are adopted to discuss the proposed anomaly detection method. The details of SHM contents and corresponding sensors are listed in Table 1. The original sampling frequency of the five types of sensors is 50 Hz.

### 2.3. Noise and Anomalies in SHM Data

**Figure 4:**
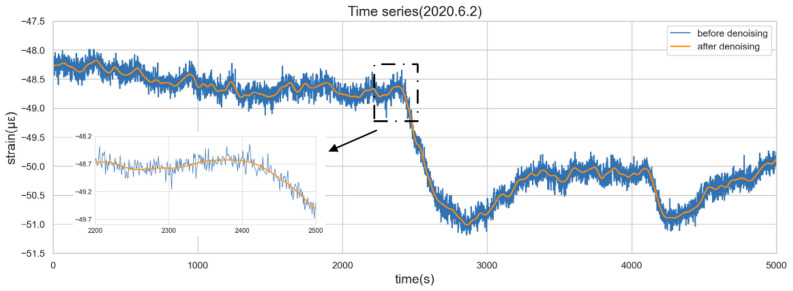
Time series before and after denoising.

In the process of data acquisition, the SHM system will inevitably be affected by environmental noise, the unstable connection between devices, an aging electric grid, unstable wireless transmission speed, channel faults, and so on, which will result in poor data quality. Figure 4 displays an enlarged picture of the concrete strain data. The burr signals can be seen in the sequence, and the changing patterns of data are easily submerged in noise.

The wavelet threshold denoising method is adopted to eliminate the noise. This study selects the optimal wavelet basis and the number of wavelet decomposition layers by applying the method of calculating the SI index mentioned in the author’s previous paper [14]. In particular, by applying the basis wavelet (Symlet 12) to decompose the original data into five layers, a string of appropriate coefficients and detail coefficients are obtained. Then, a fixed threshold method is used to calculate the wavelet threshold, which is a common method for setting the wavelet threshold in wavelet analysis. The formula is $T=\sqrt{2\log\left(N\right)}$, where N is the signal length. The detail coefficients lower than the threshold are viewed as meaningless noise and set to zero so that the noisy part of the data is removed. Finally, the denoising sequence is reconstructed by an inverse wavelet operation using the detail coefficients of each layer and the appropriate coefficients of the fifth layer.

Figure 4 shows the time series before and after denoising. It can be seen that the wavelet denoising can effectively remove the redundant information brought into the system by the external environmental noise, which helps reveal the true characteristics of the sequence. 

It should be noted that the denoising procedure does wipe off the point anomalies (classified in Section 2.4) within the sequence. These point anomalies can be easily spotted by setting thresholds according to moving windows. However, a previous study has pointed out that anomalies in SHM data not only come from poor data quality but are also caused by structural damage [31]. In this study, it is assumed that the point anomalies only refer to sources of poor data quality because the immersed tunnel was newly built and in good condition. Therefore, such anomalies can be marked down before the denoising process. The above denoising method serves the purpose of removing the interferential noise in the original data and thus improving its intelligibility and analyzability. The denoised data are ready for anomaly detection, especially with contextual anomalies and collective anomalies.

### 2.4. Classification of Anomalies

**Figure 5:**
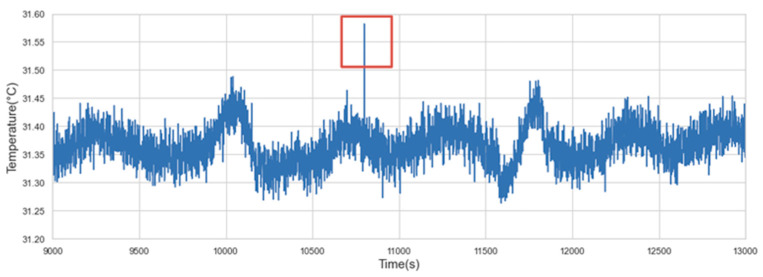
Point anomaly.

**Figure 6:**
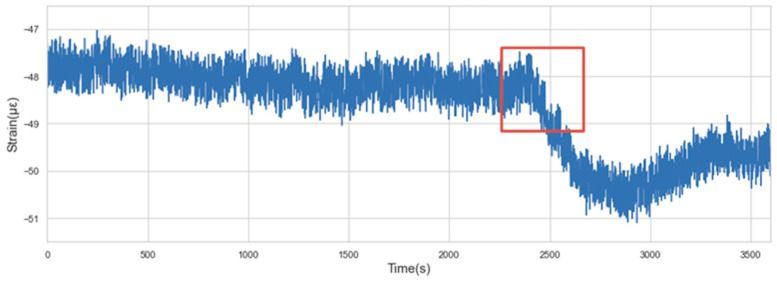
Contextual anomaly.

**Figure 7:**
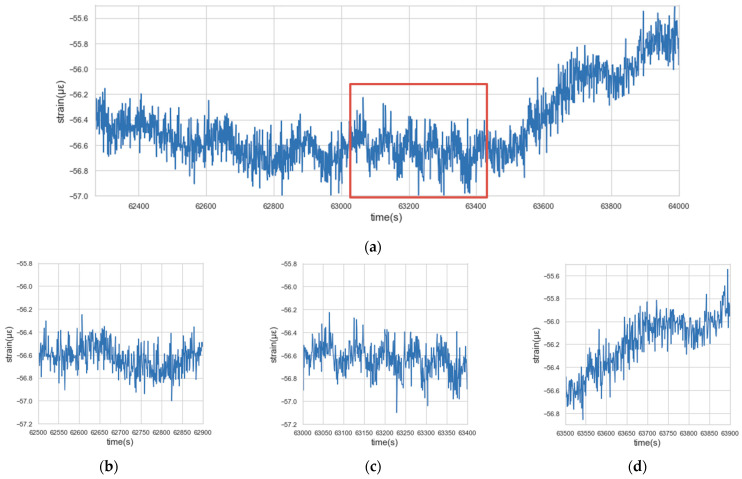
Collective anomaly: (**a**) Collective anomaly (a global view); (**b**) Before the anomaly; (**c**) The anomaly; (**d**) After the anomaly.

Anomalies refer to data deviating from the normal behavior of data. According to the characteristics of the anomalies, they can be divided into the following three categories:

(1) Point anomaly: If an individual data instance differs greatly from other data, it will be regarded as a point anomaly. The maximum and minimum values in the statistical distribution may likely be considered point anomalies. Figure 5 shows an example of a point anomaly in the structural temperature data. It can be seen that the value in the red box is much higher than the other data.

(2) Contextual anomaly: If an individual data instance differs greatly from its nearby data or within a certain context, it is called a contextual anomaly. Figure 6 shows a contextual anomaly in the concrete strain data. It can be seen that the value in the box has a downward trend against the context where the previous sequence trend is relatively stable.

(3) Collective anomalies: Collective anomalies refer to the situation where a collection of related data instances is anomalous, while the individual instance within the collective anomalies may not be anomalous by themselves. In other words, the collective anomalies happen only in the form of a collective group. Figure 7 shows the collective anomalies in the concrete strain data. In Figure 7a, the frequency of the data instances within the red box is significantly higher than that before or after the red box. Meanwhile, judging from Figure 7b–d separately, there is no anomaly detected.

In the three types of anomalies discussed above, point anomalies can be identified by setting appropriate thresholds based on the statistical parameters of the data within a certain time window. However, for the recognition of contextual and collective anomalies, both the temporal and spatial character attributes need to be considered, and the method of setting thresholds may be insufficient, so more complex methods need to be proposed.

## 3. Dynamic Warning Method

### 3.1. Static ARIMA Model

#### 3.1.1. Introduction of ARIMA

The ARMA model is a widely used classical time series prediction model. ARIMA contains both an autoregression part and a moving average part so that it can both reflect the memory of the system and the noise entering the system. The ARMA model is usually written as ARMA (p, q), where p is the autoregression (AR) parameter, indicating the number of lag observations, and q is the moving average (MA) parameter, indicating the moving average window length. The general form of the ARMA model is as follows:

(1) $$X_{t}=\varphi_{1}X_{t-1}+\ldots +\varphi_{p}X_{t-p}+\varepsilon_{t}-\theta_{1}\varepsilon_{t-1}-\ldots -\theta_{q}\varepsilon_{t-q},$$

where $X_{t}$ is the measured value at period *t*, $\varepsilon_{t}$ is the error terms, $\varphi_{p}$ and $\theta_{q}$ are the parameters of the autoregression part and the moving average part.

A backshift operator *B* is defined as shifting the sequence backward by one time period. $B^{k}$ performs the operation to shift the sequence backward for *k* periods.

(2) $$B^{k}X_{t}=X_{t-k}$$

Then, Formula (1) can be equivalently written as:

(3) $$\Phi_{p}\left(B\right)X_{t}=\Theta_{q}\left(B\right)\varepsilon_{t}$$

where

(4) $$\Phi_{p}\left(B\right)=1-\varphi_{1}B-\varphi_{2}B^{2}-\ldots -\varphi_{p}B^{p}$$

(5) $$\Theta_{q}\left(B\right)=1-\theta_{1}B-\theta_{2}B^{2}-\ldots -\theta_{q}B^{q}$$

The ARMA model is only applicable to simulate stationary time series. If the time series is non-stationary, the differencing method is commonly used to turn the non-stationary sequence into stationary ones. For time series data, “stationary” means that the mean of the data can be considered to be statistically invariant, while “non-stationary” means that the mean of the data cannot be considered to be statistically invariable [32]. The ARMA model with a differencing process is also called the ARIMA model. “I” stands for integrated, which signifies that AR and MA techniques are combined into a single model.

We define the differentiated series as the difference between the current period and the prior period:

(6) $$\nabla X_{t}=X_{t}-X_{t-1}$$

The difference operator ∇=1−B is introduced here, and the ARIMA model of order (p, d, q) can be expressed as:

(7) $$\Phi_{p}\left(B\right)\nabla^{d}X_{t}=\Theta_{q}\left(B\right)\varepsilon_{t}$$

where d is the number of times that a raw sequence is differenced, indicating the number of differences needed to be stationary.

#### 3.1.2. Time Series Pre-Processing

If the time series is non-stationary, statistical models based on historical data cannot predict the future. If the sequence is white noise, which means there is no correlation between the values of the sequences, the sequence is random and has no value for analysis. Therefore, the time series suitable for the ARIMA model must both be stationary and non-white noise after difference. This section will discuss the stationarity and randomness of time series and conduct necessary data pre-processing.

**Figure 8:**
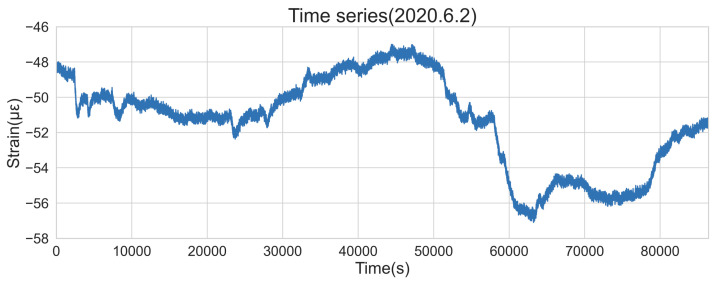
Timing diagram of concrete strain data on 2 June 2020.

Considering that ARIMA is suitable for short-term prediction, and the historical data used for modeling is generally less than 10 (that is, the value of p and q is generally less than 10), the paper takes 100 observations (100 s as the time window) to fit the model. It should be noted that the time window of 100 s is obtained subjectively after many attempts by considering the prediction accuracy of the model and the significances of many statistical tests during the modeling procedure. It is not necessarily the best model for all time points. The denoised concrete strain data of the immersed tunnel of the HZMB on 2 June 2020 is taken as an example for modeling, as shown in Figure 8. 

The timing graph of the initial series and differenced series, ACF diagram, and PACF diagram are shown in Table 2. There is no specific trend, amplitude, or frequency change in the timing diagram of the second-order difference sequence, so it is tentatively judged that the sequence has met the requirements of stationarity.

The ADF test was used to conduct a statistical test on the data, as shown in Table 3. It can be seen that the original sequence is not stable with the first-order difference sequence, and the second-order difference sequence is stable in the statistical sense with a *p*-value of 0.0045. Therefore, the order of d is determined to be two.

The Ljung-Box (LB) test can be used to determine whether a sequence is white noise. The LB test assumes that the test sequence is a white noise sequence, so the correlation coefficients between time steps are zero, and the test statistics should obey the Chi-square distribution. If the calculated *p*-value is greater than 0.05, it is 95% sure of rejecting the null hypothesis and judging that the sequence is not a white noise sequence. The *p*-value of the second difference sequence is $2.29\times 10^{-22}$, indicating that the sequence is not white noise and is worthy of further analysis.

#### 3.1.3. Model Identification

The next step is to set the number of AR and MA terms, also called model identification. A common method is to visually inspect the autocorrelation function (ACF) plot and partial autocorrelation function (PACF) plot. Note that for the second difference sequence (Table 2), the ACF plot decays slowly, while the PACF plot only has a significant spike at lag 2. This indicates that the autocorrelation pattern of the sequence is more clearly explained by adding AR terms instead of MA terms, and the lag at which the PCAF cuts off is the appropriate number of AR terms. 

However, it is not feasible to select the p and q parameters manually in the context of dynamic modeling. In this paper, the Akaike Information Criteria (AIC) and Bayesian Information Criteria (BIC) are adopted to select the p and q automatically. To be specific, model identification was carried out on the data at different times of the day, each with a 100 s time window. Table 4 shows the information criteria values under a range of combination of p and q parameters ranging from 0 to 10. According to the two criteria, most of the data performed best when (p, d, q) are set as (5, 2, 0).

This result is logical. On the one hand, setting the number of AR terms as 5 indicates that the second difference sequence still has strong autocorrelation, which is consistent with the ACF figure in Table 2. On the other hand, the wavelet threshold denoising implemented in the previous chapter filters out the effect of residuals so that the order of MA is 0. It should be noted that due to the time-consuming process of model identification, (p, d, q) is selected as (5, 2, 0) in this paper to reduce the modeling time. Therefore, the model in this paper can be expressed as:

(8) $$\nabla^{2}X_{t}=\varphi_{1}\nabla^{2}X_{t-1}+\varphi_{2}\nabla^{2}X_{t-2}+\ldots +\varphi_{5}\nabla^{2}X_{t-5}+\varepsilon_{t}$$

#### 3.1.4. Parameter Estimation

**Figure 9:**
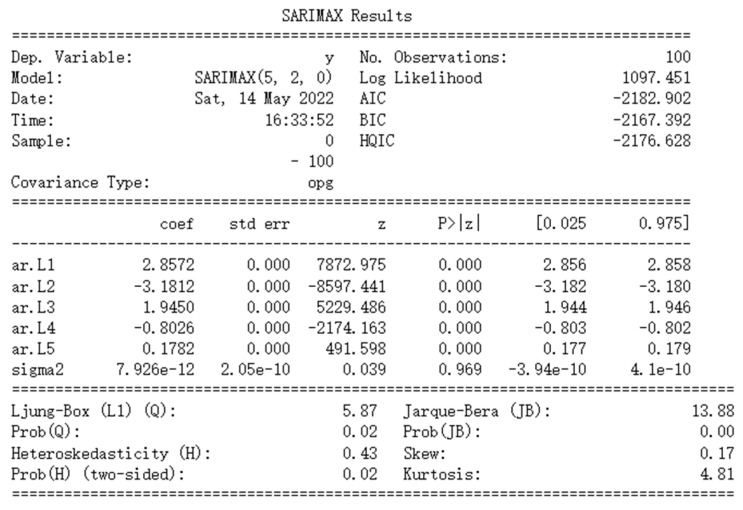
Static ARIMA result.

Maximum likelihood estimation is selected for parameter estimation. It is suitable for estimation with large samples as the idea of this method is that the current training sample distribution can represent the population distribution. Figure 9 shows the results of parameter estimation. Among them, coef. Is the list of the model coefficients, where ar.L1 to ar.L5 correspond to $\varphi_{1}$ to $\varphi_{5}$ and sigma 2 corresponds to $\varepsilon_{t}$ in Equation (8).

#### 3.1.5. Model Checking

First, check whether each term in Equation (8) has a real effect on the dependent variable. In Figure 9, the column shows that for all AR coefficients, the probabilities that the null hypothesis (no significance) is true are close to zero, verifying that each AR parameter is significant. However, for sigma 2, the probability of the null hypothesis being true is much greater than zero. This is because the absolute value of its coefficient is too small. Since the influence of sigma 2 on the dependent variable can be ignored, the non-significance of this parameter will not affect the overall model performance.

**Figure 10:**
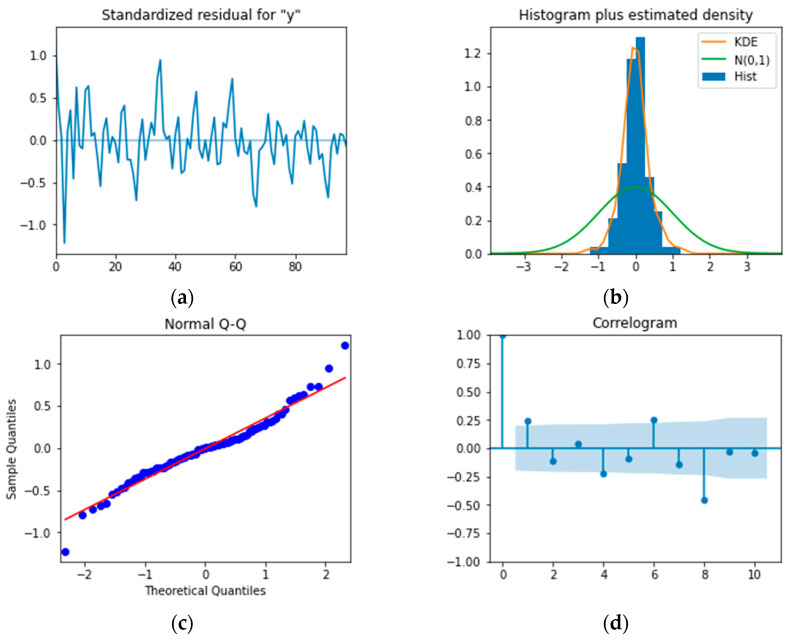
Model checking plots: (**a**) Timing graph of the residual; (**b**) Distribution plot of the residual; (**c**) Q-Q plot of the residual; (**d**) ACF of the residual.

Then, the model’s significance should be examined. The well-fitting model can extract the information contained in the time series, so its residual sequence should be close to the white noise sequence without any specific patterns. Figure 10a shows the timing diagram of the residual sequence, which exhibits strong randomness. Figure 10b,c are a residual distribution plot and quantile-quantile plot, respectively, indicating that the residual basically presents the characteristics of a normal distribution. Figure 10d is the autocorrelation plot of the residuals, which shows there is no obvious autocorrelation between the residuals. In addition, LB statistics were used for the statistical test, and the *p*-value was $5.1698\times 10^{-5}$, much smaller than 0.05. All of these verify that the residual could be considered as a white noise sequence. Therefore, the model is significant.

#### 3.1.6. Model Forecast

**Figure 11:**
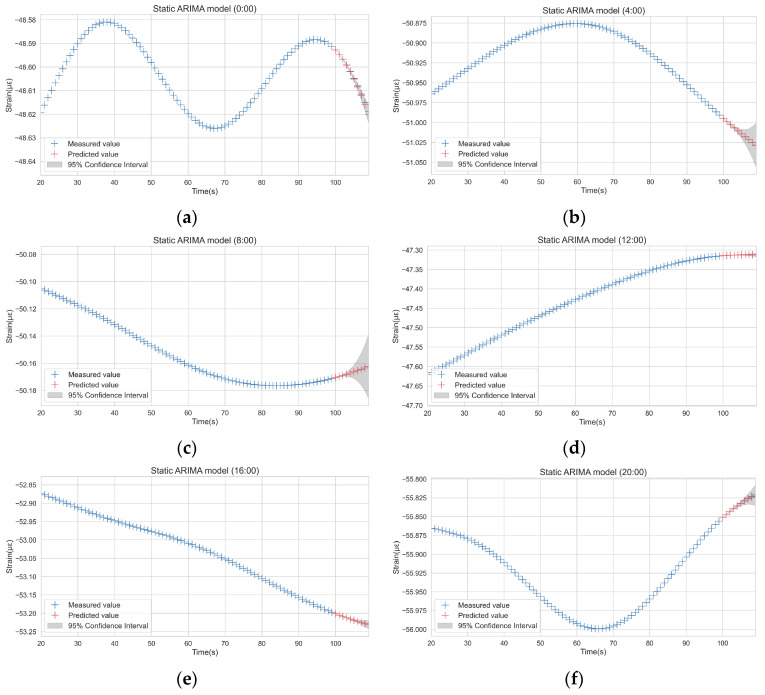
ARIMA prediction at different time periods: (**a**) Forecast results at 0:00; (**b**) Forecast results at 4:00; (**c**) Forecast results at 8:00; (**d**) Forecast results at 12:00; (**e**) Forecast results at 16:00; (**f**) Forecast results at 20:00.

The data at different times of the day are selected for static ARIMA modeling. Figure 11 shows the point prediction and prediction intervals of the model performing a ten-step forecast. It can be seen that within five time steps, ARIMA’s prediction results are good, but with the extension of the prediction period, the accuracy gradually decreases.

This is because, in essence, multi-step prediction is based on single-step prediction, which means the single-step prediction is used as an input for multi-step prediction. Therefore, the error in single-step prediction will accumulate in each subsequent prediction, resulting in inaccurate multi-step prediction results. For a SHM system, real-time monitoring data are available. In order to improve the accuracy of model prediction and improve the reliability of anomaly detection, the ARIMA algorithm will obtain the measured values in real-time so as to realize a rolling single-step forecast.

### 3.2. Dynamic ARIMA Model

Apparently though, as time goes by, the ARIMA model fitted on the previous data will no longer be suitable for the prediction, and the model parameters need to be updated. A crude way to perform this is to re-create the ARIMA model at each second, but it will be too time-consuming. Moreover, the model has high accuracy in a certain period, so there is no need to update parameters second by second. Therefore, the model error monitoring method will be adopted in this study to determine when the model requires being updated. Specifically, the system will keep track of all observations and predictions in the last 10 min. When the average error, which is the difference between the observations and the predictions, is greater than a certain threshold, the model is considered to be inaccurate. The system will automatically update the parameters based on the latest 100 s observations. Meanwhile, in order to improve the efficiency of model refitting, the parameters trained at the previous moment are taken as the initial parameter of the new model to reduce the time for parameter searching. In other words, the model will update based on its actual fitting performance. The mechanism of the dynamic ARIMA model is shown in Section 3.3.

**Figure 12:**
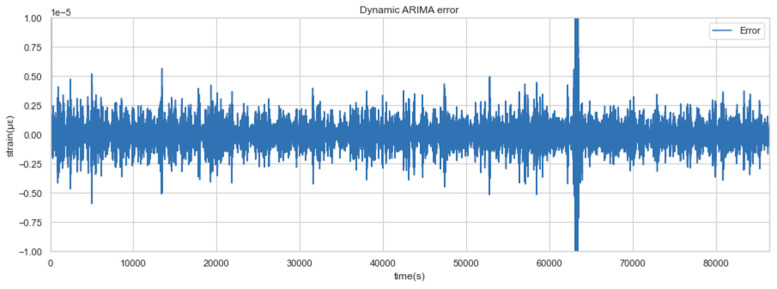
Dynamic ARIMA error sequence.

The test results show that when the update threshold is selected as $5\times 10^{-8}$, the model is updated 29 times in one day and the error sequence fluctuates steadily within a day, as shown in Figure 12. The fact that errors do not increase significantly over time indicates that the model error monitoring method can ensure the accuracy of the dynamic model.

### 3.3. Anomaly Detection Method

In this section, this study will design an anomaly detection method based on dynamic model prediction. It should be noted that the method does not involve a multi-step prediction but a rolling single-step prediction by constantly rolling forward the time window and adding the observations into the input of the model.

If the observation deviates too far from the prediction, it is considered to violate time continuity and will be identified as an anomaly. The warning threshold here should be dynamically selected according to the state of the external environment and tunnel structure. Inspired by PauTa Criterion [33,34], or the 3 σ rule, the threshold can be set according to historical data in a certain period. As the judgment is based on statistical characteristics of the data, it requires that the sample data are approximately normally distributed, so the referential period should be sufficiently long. Moreover, it is accurate to set a fixed threshold for evaluation only under stable conditions, so the reference period should not be too long. After comprehensive consideration, the reference period is set as one hour. It is assumed that the system state is relatively stable within two hours, so the threshold of this hour can be set based on the standard deviation of the previous hour.

**Figure 13:**
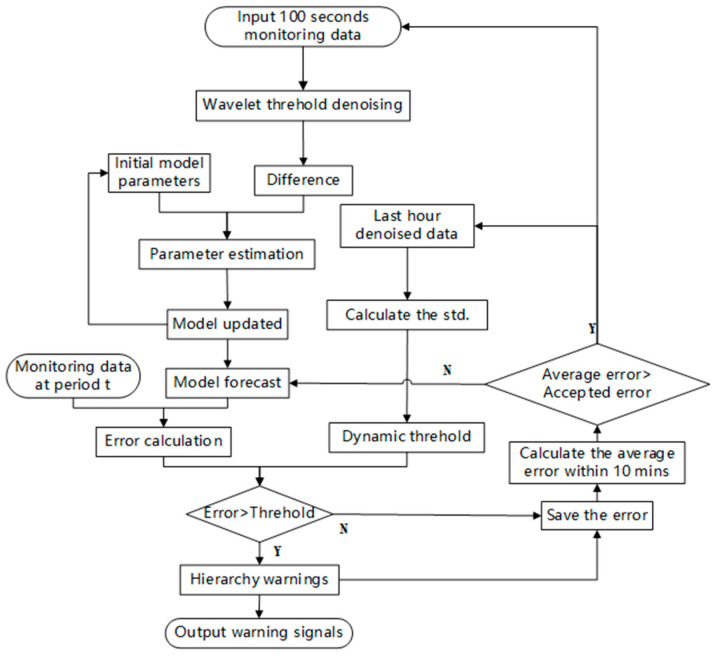
Flow chart of the anomaly detection process.

The following flow chart in Figure 13 summarizes the whole process of anomaly detection based on a dynamic ARIMA model.

**Figure 14:**
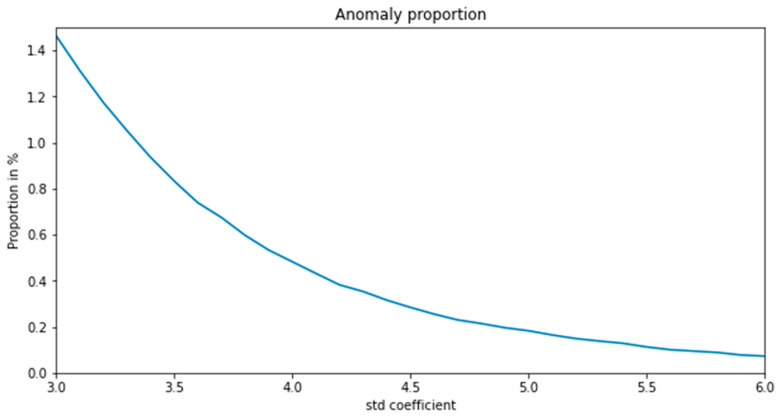
Anomaly proportion under different std. coefficients.

As shown in Figure 14, when the threshold takes different standard deviation coefficients, the proportion of anomalies recognized changes. When the std. coefficient is larger, the threshold is larger, which means the criteria are stricter, so the number of detected anomalies is smaller.

In order to adapt corresponding treatments to the anomalies with different severity, hierarchical warnings are defined by classifying the anomalies by their detection thresholds, as shown in Table 5. The std. coefficients for these three levels are defined according to the percentage of abnormality in the total data; 5.5, 6.5, and 7.5 times correspond to 0.1%, 0.05%, and 0.02%, respectively. As the std. coefficient goes up, they are labeled as Level 3 to Level 1 warning points, with Level 1 being the most urgent one.

**Figure 15:**
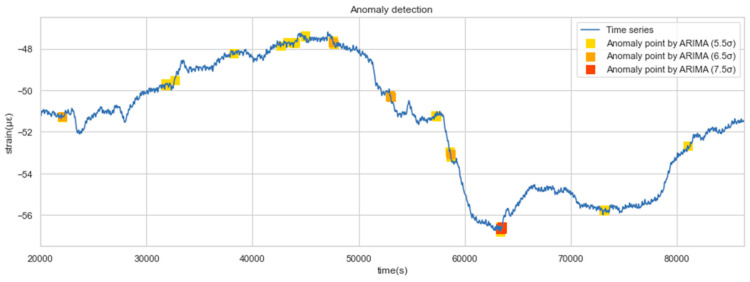
Anomalies of different levels.

Figure 15 shows the detected anomalies with the above three levels of thresholds. The model gives warning signals at both daily minima and maxima. As the data are changing more dramatically, the warning level is more severe. It should be noted that there are many physical causes of a warning identified by the proposed method, such as the drastic changes in the surrounding environment of the tunnel, the failure of monitoring sensors, or the abnormal conditions of the tunnel structure. Since the actual conditions corresponding to the abnormal signals have not been identified one by one, we can only inform the operation and maintenance authorities that they should pay close attention to the further development of the monitoring value and be prepared for possible structural defects.

It can be counted in Figure 15 that there were more than 10 anomalies in a single day, which may be too many in practice. We have acknowledged that the identified anomaly does not necessarily represent structural damage, it could just be an overweight vehicle passing through a tunnel, or sensor drift errors. In order to avoid ineffective work caused by handling excessive warnings, we give the following recommendations for anomaly detection of SHM data: in a certain time period, the number of warnings should vary within a certain range. If the number of warnings identified on a given day is significantly higher than that in the past few days, it can be assumed that some problems may really have happened, which requires a comprehensive and detailed inspection of structures, sensors, etc.

## 4. Conclusions

This study introduces specific processes of data anomaly detection based on the dynamic ARIMA model. The conclusions are drawn below:

(1) Based on the analysis of concrete strain SHM data of the HZMB immersed tunnel, three types of anomalies can be classified and should be detected. In addition, the classification of data anomalies caused by poor data quality and structural damage requires further study.
(2) The static ARIMA model is established according to the normative steps, and the model is tested to ensure its validity.
(3) Considering the requirement of real-time warning of the SHM system, the method of dynamic modeling and setting dynamic threshold value is discussed. It is suggested to adopt the multiple standard deviations of the previous time period as the dynamic threshold.
(4) A dynamic warning schematic was established with a hierarchical grading standard, from Level 1 to Level 3 warnings, to verify and apply to detect anomalies of the concrete strain data of the HZMB immersed tunnel. It is found that the proposed method is able to give good results in anomaly detection and greatly improve the efficiency of tunnel operators, which demonstrates its ability to be applied to major infrastructure structural health monitoring.

## Figures and Tables

**Table 1:** SHM contents and corresponding sensors.

Monitoring Items	Data	Sensors
Structural responses	ground motion	3D accelerometer
	strain of element	FBG strain sensor
	joint deformation	displacement meter
Environmental loads	temperature	thermometer
	humidity	hygrometer

**Table 2:** Timing graph, ACF, and PACF of differenced series.

Type of Plots	Initial Series	First Difference Series	Second Difference Series
Timing graph	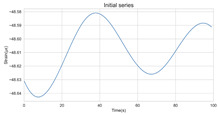	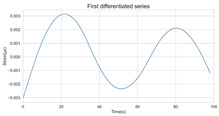	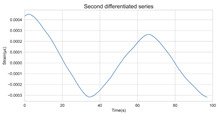
ACF plot	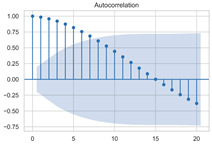	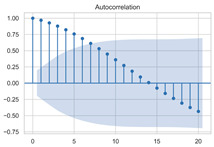	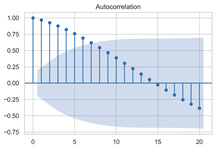
PACF plot	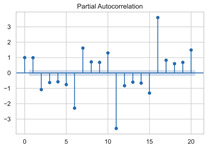	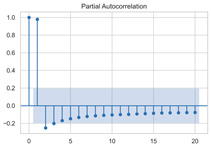	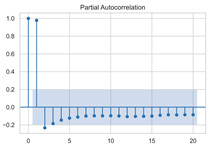

**Table 3:** ADF test results.

Series Type	Test Statistic	5% Critical Value	*p*-Value	Test Results
Initial	0.1798	−2.8950	0.9711	Non-stationary
First difference	−1.1433	−2.8958	0.6975	Non-stationary
Second difference	−3.6732	−2.8962	0.0045	Stationary

**Table 4:** Best (p, d, q) selection.

Test Data	AIC	BIC
data [0:100]	5, 2, 0	2, 2, 0
data [10,000:10,100]	5, 2, 0	5, 2, 0
data [20,000:20,100]	5, 2, 0	5, 2, 0
data [30,000:30,100]	5, 2, 0	5, 2, 0
data [40,000:40,100]	5, 2, 0	5, 2, 0
data [50,000:50,100]	2, 2, 0	2, 2, 0
data [60,000:60,100]	1, 2, 1	1, 2, 1
data [70,000:70,100]	5, 2, 0	2, 2, 0

**Table 5:** Warning level setting.

Std. Coefficient	Warning Level	Colors *
5.5	Level three	Yellow
6.5	Level two	Orange
7.5	Level one	Red

* The color of warning level intuitively shows its urgent level. Red is the most urgent situation, followed by orange and yellow.

## Data Availability

Data are available on request to the authors.
